# Mental health and psychological wellbeing of maritime personnel: a systematic review

**DOI:** 10.1186/s40359-022-00850-4

**Published:** 2022-05-30

**Authors:** Samantha K. Brooks, Neil Greenberg

**Affiliations:** Department of Psychological Medicine, King’s College London, Cutcombe Road, London, SE5 9RJ UK

**Keywords:** Maritime health, Maritime personnel, Mental health, Occupational health, Seafarers, Wellbeing

## Abstract

**Background:**

Seafaring has frequently been reported to be a ‘risky occupation’ in terms of both physical and mental health. Individuals working in seafaring professions are exposed to various stressors in the workplace, including social isolation, exposure to poor physical conditions and long work hours. This systematic review aimed to update previous reviews by collating recent literature (published between 2012 and 2021) on the factors associated with mental health and wellbeing in seafaring personnel.

**Methods:**

Four electronic databases were searched in April 2021 for primary peer-reviewed studies on factors associated with the mental health and psychological wellbeing of seafarers or interventions to improve the wellbeing of seafarers, published in English in or after the year 2012. Thematic analysis was used to synthesise the data and standardised measures of quality appraisal were used to assess risk of bias.

**Results:**

Sixty-three studies were reviewed. Risk factors for poor mental health among seafarers appear to be younger age; being single; poor physical health; exposure to noise/vibration; feeling unsafe; high job demands; long working hours; night/irregular shifts; poor sleep; poor team cohesion; poor perception of management; poor social support; lack of autonomy; scheduling uncertainties; long duration at sea; and over-commitment.

**Conclusions:**

There are numerous steps that maritime managers could take to improve the wellbeing of their personnel, including increased monitoring of the potential for poor mental health in their staff, increasing crew numbers and provision of education and support.

## Background

Working onboard a ship often involves considerable mental and physical demands which are not readily comparable to those experienced within onshore professions [1]. Seafarers have a relatively unique role in that they are in the workplace during both working and non-working hours with only their colleagues for company, making them an extremely isolated working group [2]. As they spend so much time with their colleagues, it is important that these relationships are positive and that there is cohesion amongst teams; however, research suggests there are frequently conflicts between different ranks and departments which, coupled with long periods away from home and families, can lead to loneliness and homesickness [3, 4]. Seafarers are also typically isolated in a physical environment which is not optimal for mental health: being on board a ship can involve prolonged exposure to poor physical conditions such as high-pitched noises, vibration, cold spells, high temperatures, and unstable moisture conditions [5].

Additionally, seafarers typically work long hours doing physically demanding work with inadequate rest hours; the most recent Seafarers Happiness Index report [6] revealed that many seafarers feel pressured to work excessive hours. Seafarers typically work on a ‘watch system’ which can reduce their amount and quality of sleep leading to fatigue, which can be further exacerbated by the different time zones during long voyages [7]. Other risk factors for fatigue include disrupted Circadian rhythms caused by shift work; long shifts; irregular work hours; a rotating watch system rather than steady watch cycle; night shifts; irregular sleep quantity; high job demands and pressures; and exposure to physical environment factors such as ship engine noise and vibration [7–10].

Previous reviews of seafarers’ mental health have examined: factors associated with stress [1, 5, 7, 11, 12]; maritime pilots’ wellbeing and job satisfaction [13] and depression and suicide in seafarers [14]. Collectively these have identified various risk factors associated with poor wellbeing, including loneliness and long-term separation from family and home; fatigue; high workload; long voyages; long working hours; rotating watch systems; short ship-turnaround times; little advance warning of being required for duty; environmental stressors on board such as motion, noise and vibration; economic pressure; disturbed sleep; night shifts; variable weather; limited time for recreation; lack of shore leave; lack of job security; experiencing piracy; criminalisation of seafarers and treatment of maritime incidents as ‘true crimes’; and being constantly confined on board with colleagues, often in multi-national crews with different values, expectations, understandings and languages, which can cause conflicts and poor relationships.

At the time of writing this review in the first half of 2021, the COVID-19 pandemic has posed particular challenges to the seafaring population, including how to manage infection cases on board, how to reduce the spread of disease on a ship, how to handle quarantine and testing of seafarers, interaction with shore staff in ports, crew changes, and reduced possibilities for shore leave [15]. In December 2020, the International Labour Organization’s (ILO) Committee of Experts ruled that governments had failed in their duty of care to seafarers during the pandemic, not meeting the minimum standards for basic rights such as healthcare, repatriation, annual leave and shore leave as set out in international law [16]; it has also been reported that during the peak of the pandemic, the ISWAN seafarers’ helpline saw a threefold increase in number of cases [17]. Therefore, it is particularly important now to understand the risk factors for poor mental health of those in maritime organisations and how maritime staff can best be supported.

This study aimed to update previous reviews to provide a comprehensive answer to the research question, ‘which factors are associated with mental health and wellbeing in maritime personnel?’, and also explore any literature relating to mental health interventions within the seafaring population. Of the previous reviews discussed, only two were done systematically [11, 13]. The former explored stress in seafaring professions in the literature until 2012 whilst the latter reviewed papers published pre-2015 focused on maritime pilots only. Therefore, the decision was made to limit this review to papers published in or after 2012, to avoid duplication of earlier reviews.

## Method

### Search strategy

Embase, Medline, PsycInfo and Web of Science were searched from inception to 1^st^ April 2021 using the following search strategy: “(mental health or wellbeing or well-being or depression or anxiety or stress or resilien* or alcohol misuse or alcoholism or hazardous drinking or problematic drinking) AND (seafarer* or seafaring or sea-farer or sea-faring or Navy personnel or Marines or maritime or sailor* or seamen or seaman or mariner*)”.

### Selection criteria

Studies were eligible for inclusion if they: presented results of primary, peer-reviewed research; were written in English; were published in or after 2012; and contained data on factors associated with the mental health and psychological wellbeing of seafarers or interventions to improve the wellbeing of seafarers. Studies of seafaring military populations were included only if they considered non-operational wellbeing on board and did not focus specifically on operational demands and combat. Similarly, studies which focused only on psychological responses to traumatic experiences (such as piracy) were excluded.

### Screening

All citations were downloaded to EndNote© reference management software (Thomson Reuters, New York). One author (SKB) carried out the screening. First, titles were screened and any obviously not relevant to the review were excluded; next, abstracts were screened, again with any not relevant to the review excluded; and finally, full texts of remaining citations were obtained, and the papers were read in their entirety to ascertain whether they met all inclusion criteria. Any queries or uncertainties about exclusion were discussed with the other author (NG).

Although the review aimed to include only literature from 2012 onwards, we searched databases from inception in order to ensure that older literature had in fact been included in previous reviews. We screened the titles and abstracts of the pre-2012 studies and selected a random sample of 25 pre-2012 studies which were deemed to meet all of our other inclusion criteria. We then checked the reference lists of existing reviews to confirm that these studies had in fact been previously reviewed. As they did appear in the existing reviews, we then removed all pre-2012 studies.

### Data extraction and synthesis

The following data were extracted by SKB from each paper: first author; year of publication; country of study; design; number of participants; job role of participants; age and gender of participants; measures used; and key results. Factors predictive of mental health and wellbeing in seafarers were grouped into a typology using thematic analysis [18] on the results of the studies. This process involved coding the data (for example, any data relating to amount or quality of sleep was coded as ‘sleep’); identifying similarities between codes; and grouping codes into analytic themes (for example, codes relating to the noise, temperature, light and movement on ships were all included together within the analytic theme ‘physical working and living conditions’).

### Quality appraisal

Each of the included papers underwent individual quality assessment by SKB. Quantitative studies were appraised using a slightly modified version of the AXIS tool [19] which consists of twenty questions assessing studies in terms of their objectives, various aspects of methodology, results, discussions and conclusions. Two questions were modified so that a ‘yes’ response would be indicative of better quality, in line with the other eighteen questions (for example, the question ‘does the response rate raise concerns about non-response bias?’ was reworded to ‘was the response rate clearly reported and at least 50%?’). This enabled us to simply add up all ‘yes’ responses and give each study a total score, which was converted to a percentage of positive responses, with a higher score reflecting a higher quality paper.

Qualitative studies were appraised using a slightly modified version of the Critical Appraisal Skills Programme Qualitative Checklist [20], a ten-item quality appraisal tool assessing the methodology, data analysis and discussion of implications of qualitative studies. One question, ‘how valuable is the research?’, was reworded to ‘do the authors discuss the value of the research in terms of implications and contribution to literature?’ to allow yes/no responses in line with the other items. Again, this allowed us to add up all ‘yes’ responses to give each study an overall quality score percentage. Studies using retrospective analysis of existing health data were appraised using the nine questions in the MetaQAT Critical Appraisal Tool [21]. For mixed-methods studies, one of the quantitative or qualitative appraisal tools was used, depending which type of data was the main focus of the paper.

## Results

**Fig. 1:**
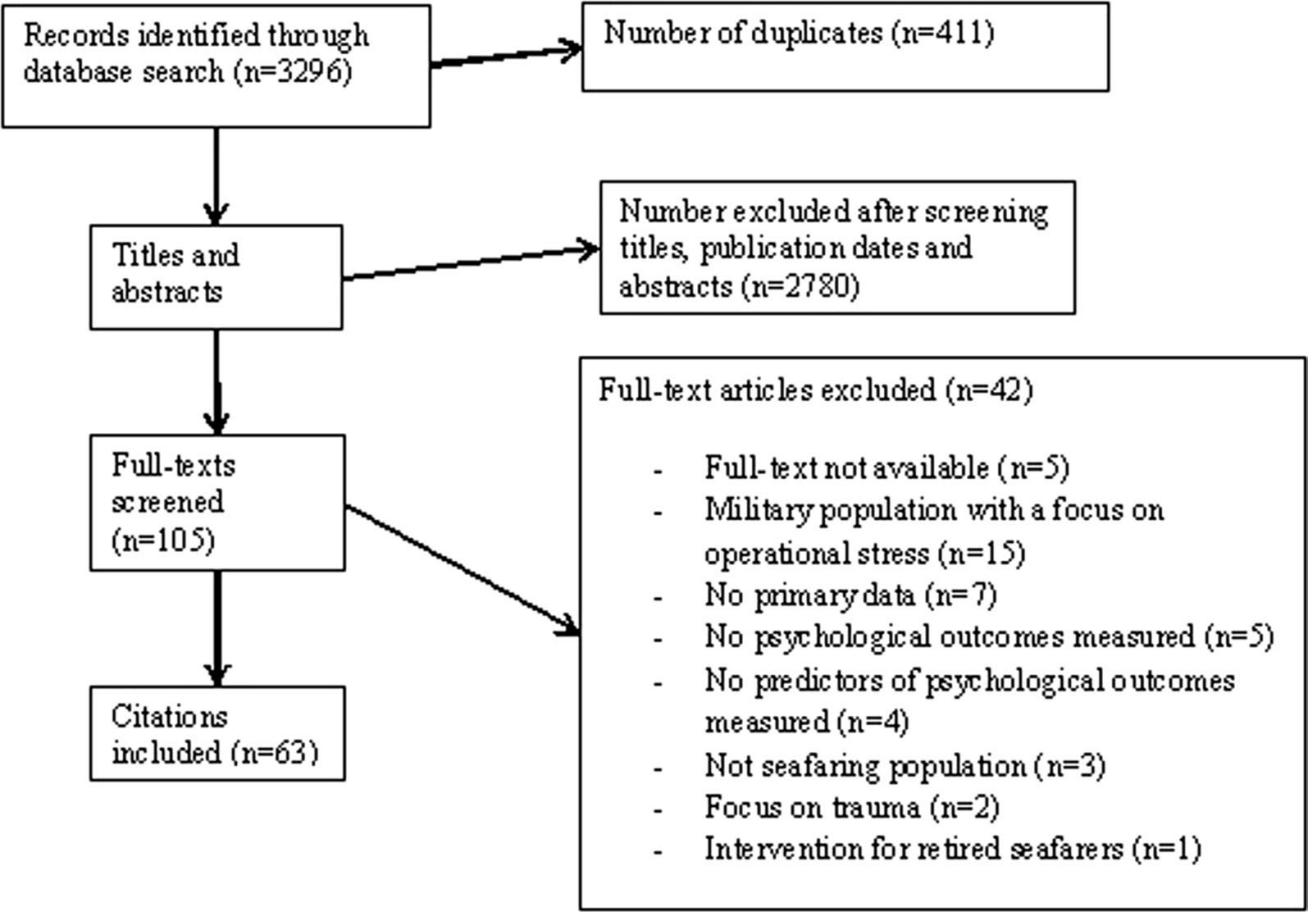
Screening process

Combined database searches produced 3,296 results, which were downloaded to EndNote© where 411 duplicates were removed. A total of 2,473 papers were excluded based on title or date of publication and 307 based on abstract. A further 37 papers were excluded after reading the full texts; additionally, five papers had no full text available, so these were also excluded. This left 63 papers included in the final review. A flow diagram of the screening process is presented in Fig. 1.

Of the 63 included studies, the majority (n = 52) were quantitative. Seven were qualitative studies, two were retrospective data analyses, and two involved both quantitative and qualitative measures. Study population sizes ranged from 22 to 917. Due to the nature of seafaring work, many of the studies included participants of multiple nationalities and from multiple countries; in terms of the authors of included studies, they were based in the USA (n = 8), Germany (n = 7), Norway (n = 5), Croatia (n = 4), South Korea (n = 4), the UK (n = 4), China (n = 3), Lithuania (n = 3), Poland (n = 3), Australia (n = 2), Denmark (n = 2), Italy (n = 2), Turkey (n = 2), France (n = 1), India (n = 1), Ireland (n = 1), Malaysia (n = 1), Philippines (n = 1), Singapore (n = 1), Sweden (n = 1), Ukraine (n = 1), or multiple different countries (n = 6). Only two studies assessed mental health interventions for seafarers.

Quality of included papers was mixed, with a mean score of 66.7% (range 25–90%). Most quantitative papers scored well on questions relating to clearly defined aims, appropriateness of methodology and variables studied, and clear reporting of statistical analyses. However, many did not include power calculations to justify sample size, contained unrepresentative samples, and failed to consider non-responders. Most qualitative papers scored well on clearly defined aims and appropriateness of methodology; however, several were reduced in quality by lack of discussion about recruitment strategies and failure to consider the researchers’ own bias and influence over the data collection and analysis. Retrospective analyses scored well on descriptions of methodology and results but tended to include potentially non-representative samples and fail to provide in-depth discussion of the potential application of their results.

An overview of characteristics of the included studies are presented in Table 1.

**Table 1:** Overview of included studies

References	Country	Participants: ‘n’ and job role	Participant demographics	Relevant measures	Quality (%)
Akamangwa [66]	UK	41 Staff at a UK-based global shipping company and aboard two of its ships—34 ship crew, 7 managers	Not reported	Semi-structured interviews about experiences complying with health, safety and wellbeing requirements on board	70
Andrei et al. [67]	Australia	Unclear; study states n = 199 but also reports that 25 were members of command team and the other 125 were members of crew Seafarers working for marine operations of a large global mining company	99% male Mean age 36.65	3 items from Questionnaire on the Experience and Assessment of Work; 4 items adapted from existing research on monotony and attention capacity literature; 3 items from Work Design Questionnaire relating to decision-making authority; 4 items adapted from existing scale on perceived availability of instrumental support from colleagues and direct supervisor; 10 items developed for previous study to measure different types of fatigue; 4 items adapted from previous literature on common sleep problems	65
Andruskiene et al. [34]	Lithuania	393 Students at Lithuanian Maritime Academy studying either Marine Navigation, Marine Engineering, or Port and Shipping Management/Finances of Port and Shipping Companies/Maritime Transport Logistics Technologies	78.9% male Mean age 20.71	Pittsburgh Sleep Quality Index; Hospital Anxiety and Depression Scale	60
Arcury-Quandt et al. [25]	USA	598 Active-duty ship-assigned military personnel—US Navy and Marine Corps	71.2% male Mean age 26.3	CES-D; CAGE questionnaire and abbreviated AUDIT-C; modified Holmes-Rahe Stress Scale	70
Barbarewicz et al. [70]	Germany	60 Maritime pilots; 12 on a 1-week rotation system and 48 on 4-month rotation system	Gender not reported Mean age 48.7	RESTQ-work 27; Berlin Questionnaire; validated short version of the 'evening morning protocols'—a sleep diary; Resilience Scale RS-13	80
Bergheim et al. [31]	Norway	486 in Study 1; 594 in Study 2 Maritime workers from 3 shipping companies	Study 1: 100% male Mean age 40.8 Study 2: 99% male Mean age 40	Study 1: Norwegian offshore risk and safety climate inventory; Psychological Capital Questionnaire Study 2: As above, plus three items from Job Satisfaction Scale—short version	70
Bergheim Valdersnes et al. [43]	Norway	397 Seafarers from a Norwegian company in the offshore oil and gas industry	Gender not reported 32.9% aged between 30–39; mean not reported	Subscale of Swedish Occupational Fatigue Inventory; questionnaire based on hazard categories used in official reports from Norwegian Maritime Directorate; Psychological Capital Questionnaire; Safety Climate Questionnaire	65
Bobdey et al. [76]	India	568 Personnel posted on a capital ship of the Indian Navy	Not reported	Interpersonal Support Evaluation List	65
Brasher et al. [46]	UK	144 submariners within the Royal Navy v 144 general service controls	Not reported	Work and Wellbeing Questionnaire; GHQ-12	70
Carotenuto et al. [48]	Authors in Italy; participants were from Argentina, Bulgaria, India, Italy and Romania	162 Seafarers on board merchant ships for at least two weeks—7 tankers belonging to the same shipping company	100% male Mean age 34.9	Psychological General Well-being Index	60
Chambers and Main [82]	Australia and New Zealand	50 Maritime pilots employed across ports in Australia and New Zealand	98% male Mean age 51.42	9-item Vitality Scale from the short form Health Survey; 10-item symptoms of fatigue checklist; 10-item coping strategies checklist	60
Chowdhury et al. [78]	India, Madagascar, Philippines, Ukraine, and 'cross-regional'	615 International Transport Workers' Federation—seafarer affiliates	100% male India; 80% male Madagascar; 98.4% male Philippines; 99% male Ukraine; 88% male cross-regional Age not reported	Study-specific questionnaire establishing knowledge, attitudes and behaviour regarding HIV/AIDS, health and wellness, health-seeking or risk-taking behaviour and the role of the workplace and union in promoting health	45
Chung et al. [29]	South Korea	160 Seafarers on shipping vessels belonging to a Korean shipping company, docked at Busan Port	99.35% male Mean age 35.77	Copenhagen Burnout Inventory; Siegrist's ERI Scale; Epworth Sleepiness Scale; Emotion Regulation Questionnaire	70
Devereux and Wadsworth [77]	Authors in UK; participants were British, Polish, Romanian, Norwegian, Russian Swedish, Dutch and Filipino	37 Seafarers berthed in UK ports on 4 vessels—one offshore vessel and three chemical/product tankers	97.3% male Mean age 39	Semi-structured interviews covering seafarers’ experiences of the arrangements made to manage risks to their health and safety	80
Dohrmann et al. [49]	Denmark	193 Employees of Danish ferry shipping industry; most slept at home or in onshore watch-rooms	89% male Mean age 47.6	Swedish Occupational Fatigue Inventory; two subscales of Copenhagen Psychosocial Questionnaire (work-family conflict and supervisor support)	70
Dohrmann et al. [61]	Denmark	As above	As above	Swedish Occupational Fatigue Inventory; two subscales of Copenhagen Psychosocial Questionnaire (job demands and control)	65
Doyle et al. [27]	Authors in Ireland, participants international	387 Seafarers of tanker vessels in an international gas and crude oil shipping company who had been on board between 0–24 weeks; 1% catering, 28% rating/crew, 65% officer/engineer	98% male 21% aged 18–29, 37% between 30–39, 41%between 40–64, and 1% 65; mean not reported	2 questions each asking number of weeks participants had been on board since last shore leave and how long they have worked as a seafarer; Dispositional Resilience Scale-15; 4-item version of Perceived Stress Scale; The Employees Survey—an annual survey of work attitudes and experiences completed anonymously by employees	70
Hystad and Eid [42]	Authors in Norway, participants international	742 Seafarers working in the offshore re-supply industry (402) and seafarers working on board combined passenger and cargo ships (340)	Gender not reported Offshore supply: 12.2% aged 24 or under, 16.9% aged 25–29, 32.3% aged 30–39, 27.6% aged 40–54, 9.7% aged 55 or over Seafarers on freight and passenger ferries: Mean age 37.02	Psychological Capital Questionnaire; Swedish Occupational Fatigue Inventory; Pittsburgh Sleep Quality Index; asked to judge the extent to which they felt disturbed by different environmental factors such as noise and motion; asked how long they had been on board since last shore leave and length of seafaring career	65
Hystad et al. [30]	Authors in Norway, vessels operating in North Sea and Southeastern Asia	402 Seafarers working in offshore oil and gas re-supply industry	Gender not reported 12.2% aged 24 or under, 16.9% aged 25–29, 32.3% aged 30–39, 27.6% aged 40–54, 9.7% aged 55 or over	Swedish Occupational Fatigue Inventory; Zohar and Luria's scale of safety climate; questions on psychological demands and job control from General Nordic Questionnaire for psychological and social factors at work	65
Jegaden et al. [50]	France	80 seafarers (40 officers, 40 crew) vs 63 office staff from the same shipping company	100% male Mean age 40.3 for officers, 42.3 for crew members, 43.6 for office staff	Farmer and Sundberg Boredom Proneness Scale; Hospital Anxiety and Depression Scale; Job Content Questionnaire	45
Jezewska et al. [41]	Poland	300 Seafarers employed in the Polish fleet and foreign flag vessels	100% male Mean age 44	WHOWOL-BREF; Survey for people working at sea; NEO-FFI Questionnaire; PTS Temperament Questionnaire	60
Jo and Koh [33]	South Korea	146 officers on Navy ships, 98 officers on submarines	Ship officers: 97.5% male, mean age 30.7 Submarine officers: 100% male, mean age 29.61	Items from Korea National Health and Nutrition Examination Survey; Job-Related Affective Well-Being Scale	75
Kalvaitiene and Sencila [69]	Lithuania	45 Marine navigation, marine engineering and electrical engineering students at the Lithuanian Maritime Academy, all with seagoing practice experience	Not reported	Semi-structured interview with questions about difficulties on board; communication with ship's crew members; and measures taken to make it easier to adapt on board	30
Kelley et al. [75]	USA	108 US Navy personnel assigned to a homeported Arleigh Burke-class destroyer anticipating deployment within 2 months	70.4% male Mean age 28.15	AUDIT; Command Stress Assessment; CES-D-10; Friendship Scale Assessment; Relationship Assessment Scale	75
Kelley et al. [81]	USA	101 US Navy personnel assigned to an Arleigh Burke-class destroyer who experienced an 8-month deployment	71.3% male Mean age 28.34	Command Stress Assessment; Pittsburgh Sleep Quality Index; AUDIT; CESD-10	75
Kim and Jang [28]	South Korea	149 Marine officers working at a harbor (people in command of a commercial vessel and the crew)	100% male Mean age not reported, but half were in their 20 s and 30 s	SCL-90-R; survey on job stress modified and customised for seafarers; modified tool based on Job Descriptive Index	75
Kim and Jang [44]	South Korea	280 Seafarers of a shipping firm, living and working on a ship for more than 6 months	Gender not reported 80.4% in 20 s-30 s, 19.6% aged 40 +	Modified version of organisational culture tool; tool based on tool for emotional and instrumental support; Cho et al.'s self-efficacy measurement; short form of Park et al.'s perceived fatigue tool; modified version of Psychological General Well-Being Index (PGWBI-S)	70
Kingdom and Smith [83]	UK	282 Coastguards	76% male Mean age not reported, but 64% aged between 41–60	Questionnaire measuring exposure to physical agents and noise; job demands-control-support; effort-reward imbalance; organisational culture; management of change; leader-member exchange; team-member exchange; bullying; role conflict and ambiguity; training; how stressful work is; number of sick days in past year; work-related illness; Hospital Anxiety and Depression Scale; Epworth Sleepiness Scale; symptoms and medication; insomnia; accidents and injuries; memory; risk-taking; smoking and drinking; weight and exercise; time to relax; time spent on hobbies; impact of family on job; impact of job on family; negative affectivity; coping	60
Kum and Ertas [73]	Turkey	50 Shipping company personnel	78% male Mean age not reported, but 40% under 30, 40% between 30–40, 20% over 40	Questionnaire derived from Work Harassment Scale	60
Lefkowitz et al. [23]	Authors in USA, data international	Examined 278 mental illness claims between 2007–2015 from marine insurance provider for seafarers on Gard vessels	N/A	N/A	77.8
Lefkowitz et al. [45]	USA	233 Domestic shipping vessel masters (captains) and pilots at two vessel piloting training centres	Gender not reported Mean age 46	PHQ-9; GAD-7; Sleep Condition Indicator	65
Matsangas and Shattuck [56]	USA	892 Sailors on US Navy surface ships	78.8% male Median age 25 (mean not reported)	Pittsburgh Sleep Quality Index; Epworth Sleepiness Scale; Insomnia Severity Index; Profile of Mood States	65
McVeigh and MacLachlan [60]	Philippines	32 Merchant seafarers on board liquefied natural gas carriers, product oil tankers and crude oil tankers	100% male Age not reported	Focus groups to discuss perceptions and experiences of stress, resilience and wellbeing	80
McVeigh et al. [35]	Not reported; authors based in Ireland, South Africa, Czechia, and the UK	512 at Time0, 276 at Time1 (approximately 10 months later) Merchant seafarers within a large shipping organisation (officers and ratings/crew)	Time0: 98.2% male, 41% aged 40–64 Time1: 98.2% male, 39.9% aged 40–64	The organisation's Employees Survey; Dispositional Resilience Scale-15; Perceived Stress Scale-4	65
McVeigh et al. [68]	Not reported; authors based in Ireland, South Africa, and Czechia	24 Superintendents (office-based, n = 5), and officers and crew (n = 19) of a large shipping company	Not reported	11 interviews and 1 focus group with 13 participants The first six interviews assessed perceptions of the pilot resilience programme and perceptions and experiences of resilience Remaining interviews and focus group covered perceptions and experiences of wellbeing, resilience and stress	70
Nielsen et al. [63]	Norway	541 Seafarers from 2 large Norwegian shipping companies	99% male Mean age 40	3 items about intentions to leave; three items from Job Satisfaction Scale—short version; Brief Norwegian offshore risk and safety climate inventory (Brief-NORSCI); 5 items from Multifactor Leadership Questionnaire; Authentic Leadership Questionnaire' 3 items on job demands; Negative Acts Questionnaire Revised; 4 items from Platoon Cohesion Index adapted for maritime context	75
Oldenburg et al. [32]	Germany	251 Seafarers in merchant marine service	92.8% male Mean age 41.9	Study-specific scale of shipboard stressors; emotional exhaustion scale of the Maslach Burnout Inventory; one question each; Epworth Sleepiness Scale	75
Oldenburg et al. [58]	Germany	104 Sailors on board container ships (19 nautical officers, 51 deck ratings and 34 engine room employees)	100% male Mean age 35.4	Questionnaire used in the authors’ previous maritime studies, provided in form of a standardised interview, asking participants to rate whether they experienced various physical environmental influences and a free-text response on physical influences during their stay on board	75
Oldenburg and Jensen [38]	Germany	323 Seafarers on board container ships (nautical officers, deck ratings, engine room personnel)	100% male Mean age 38.3	Study-specific questionnaire about communication of crews with their home	70
Oldenburg and Jensen [51]	Authors in Germany, participants European and Southeast Asian	323 Sailors on container ships	100% male Mean age 38.3	Participants recorded working time, leisure time, lying/sleeping time and sport time; SenseWear armband monitor and Polar watch worn continuously to give an objective measure of strain (watch measures heart rate and heart rate variability; armband monitors physical activity and calorie expenditure); asked about subjectively experienced stress due to job-related physical or mental impacts, working hours, and sleep deficit	75
Oldenburg and Jensen [57]	Germany	323 Seafarers on board container ships (nautical officers, deck ratings, engine room personnel)	100% male Mean age 38.2	Study-specific questions on mental and physical strain as a result of activities carried out in the respective voyage episodes and various seafaring stressors, derived from their own previously published study	75
Oldenburg and Jensen [79]	Germany	323 Seafarers on board container ships (nautical officers, deck ratings, engine room personnel)	100% male Mean age not reported, median 37	Study-specific questionnaire asking about use of and needs for recreational facilities on board and strategies for coping with stress; daily log of leisure activities and sleeping time	70
Osterman et al. [22]	Sweden	1980 (survey); 29 (interviews) Survey: Service crew on passenger ships Interviews: 5 HR department, 4 hotel, restaurant and cruise managers, and 20 ratings service department including safety delegates and union representatives, and seafarers working in bars, restaurants, housekeeping, shops, warehouses and spas on board	Survey: Mostly men but exact statistics not reported; largest age group 20–30 Interviews: 51.7% male; age not reported	Survey based on questionnaires from the International Social Survey Program, Work Orientations III relating to organisational and occupational commitment, job satisfaction and work experiences Unclear what the semi-structured interview questions were	85
Othman et al. [52]	Malaysia	60 Seafarers—20 senior deck cadets, 20 senior deck officers, 20 junior deck officers	Not reported	‘Technique for Order Performance by Similarity to the Ideal Solution’ method used to rank the root causes of distractions causing accidents at work	25
Peplinska et al. [39]	Poland	210 Mariners working on deep-sea ships	Gender not reported Age range 25–60, mean not reported	Purpose In Life Test; State-Trait Anxiety Inventory; Perceived Stress Questionnaire; Questionnaire of Suitable Marriage	45
Peplinska et al. [40]	Poland	210 Mariners working on deep-sea ships	Gender not reported Age range 25–60, mean not reported	State-Trait Anxiety Inventory; Levestein's Stress Level Questionnaire; Well-Matched Marriage Questionnaire	50
Pesel et al. [64]	Authors in Spain, Italy and Denmark; participants international—54% from Asian countries, 17% from European countries, 28% from Russian and former USSR countries, 1.3% other	72 Seafarers on container ships	100% male Mean age 39	GHQ12 with three extra questions about COVID precautions on board	65
Rapoliene et al. [84]	Lithuania	Baseline: 180 (65 balneotherapy intervention v 50 music intervention v 65 controls) Completed intervention: 55 v 35 v 50 respectively Seamen who had been working at sea for more than 5 years	100% male Mean age 47.5 for balneotherapy group, 47.6 for music group, 46.2 for controls	Overall reported health, medication use, pain, mood, changes in feelings assessed in GP evaluation; General Symptoms Distress Scale; Multidimensional Fatigue Inventory; Cognitive Failures Questionnaire	75
Saitzyk and Vorm [24]	USA	Retrospective database analysis of 425 cases of self-directed violence found in records Military personnel on board US Navy aircraft carriers	61.4% male Mean age not reported, majority under 25	N/A	77.8
Schmied et al. [59]	USA	22 Active duty service members from naval commands, assigned to sea duty	77.8% male 16.7% aged 18–24, 38.9% aged 25–29, 44.4% aged 30 +	Semi-structured interviews assessing experiences of sleeping in shipboard environments	80
Seyle et al. [26]	India, Philippines and Ukraine	101 seafarers who had been held hostage by pirates in the past 10 years vs. 363 not exposed to piracy	Not exposed to piracy: India—100% male, mean age 29.46 (n = 103), Philippines – 99% male, mean age 40.34 (n = 144), Ukraine—87% male, mean age 34.85 (n = 127) (Note: it is unclear why 374 participants are described when ‘n’ is reported to be 363) Exposed to piracy: India – 100% male, mean age 37.41 (n = 44), Philippines – 100% male, mean age 40.34, Ukraine – 96% male, mean age 42.88 (n = 26)	Previous trauma exposure; 2 items about training and how helpful it was; PCL-C; CES-D; Duke Health Profile; three study-specific items about the impact of piracy on work decisions	65
Shevchenko et al. [53]	Ukraine	80 Cadets specialising in sea and river transport—students from two Maritime Academies on their first or second course (n = 40) or third/fourth (n = 40), who had undergone long-term floating practice lasting over 3 months	Not reported	Coping-Strategy Indicator; Well-being-Activity-Mood questionnaire	40
Sliskovic [65]	Author in Croatia; participants international – 57 countries represented	752 Seafarers employed in the global shipping sector	89.23% male Mean age 37.34	Questionnaire with questions on sociodemographic and work characteristics and one open question about personal experiences relating to the COVID-19 pandemic	70
Sliskovic and Penezic [55]	Croatia	530 Seafarers on cargo ships	Gender not reported Mean age 37.7	Job satisfaction scale; 10 questions on specific aspects of job; 2 open questions about sources of satisfaction and dissatisfaction	60
Sliskovic and Penezic [71]	Croatia	298 Officers on cargo ships	Gender not reported Mean age 39.16	3 questions on contract (number of months contracted to be on board, number of months at home, compliance with contract regarding changes to ship and home periods); 1 question on free and unlimited access to the internet on board; Overall Job Satisfaction scale; Satisfaction with Life Scale; 5-item version of Mental Health Inventory	75
Sliskovic and Penezic [47]	Croatia	530 Seafarers employed in international shipping	Gender not reported Mean age 37.7	Study-specific scale assessing smoking, alcohol, sleeping, exercise and diet; newly-developed instrument for stress on board; Mental Health Inventory-5	70
Tedesco et al. [36]	Authors in Italy, participants international	801 Seafarers of Italian shipping companies	94.5% male Mean age 36.4	Study-specific survey on health status and smoking/drinking/drug habits; Karasek Demand-Control-Support Questionnaire	90
Turkistanli and Sevgili [54]	Turkey	266 Undergraduate maritime students (marine transportation engineering or marine engineering)	89% male Mean age 21.43	Survey created for a previous study, on awareness of health risks, general perception of danger and discomfort in the workplace and risks of contracting diseases	55
Xia et al. [80]	China	71 Crew members of a hospital ship for approximately 3 months	100% female Mean age 32.1	SCL-90	60
Xiao et al. [62]	China	917 Crew members from Nantong Entry-Exit Inspection and Quarantine Bureau	100% male Mean age 33.5	Social Support Rating Scale; Zung Self-Rating Depression Scale; Occupational Stress Questionnaire; World Health Organization Quality of Life-BREF	65
Xue and Tang [74]	China	55 Crew members and shore management of two shipping companies	Not reported	Semi-structured interviews about experiences of implementing the International Safety Management code and the outcomes and impacts of management’s safety inspections on ships	80
Yuen et al. [72]	Singapore	202 Deck officers, engine officers and ratings from containers, dry bulk, liquid bulk, and specialised carriers	Not reported	Multifactor Leadership Questionnaire; Multidimensional Scale of Perceived Social Support; scale based on literature review assessing physical environment, task-related factor, technology-related factor, organisational factor and individual factor; PsyCap Questionnaire; burnout scale from Maslach Burnout Inventory; safety behaviour scale obtained from Lu et al	60
Zhao et al. [37]	China and two European countries (not specified)	880 questionnaires and 60 interviews Questionnaires: Seafarers from two Chinese and two European shipping companies Interviews: Seafarers and managers from two Chinese and two European shipping companies	Questionnaires: 99.1% male, mean age 36.1 Interviews: gender and age not reported	Questionnaires: Shortened version of questionnaire from Cardiff Seafarers' Fatigue Research Programme—sections focusing on job/vessel, hours of work and rest, fatigue at sea, work, sleep patterns and health-related behaviours, and travel to/from vessel Interviews covered work shift schedule, work hours, workload, sleep, on-leave, fatigue and company supports	80

The following predictors were explored in the data and are discussed as themes in this review: sociodemographic and personal characteristics; physical working and living conditions; safety; job demands and pressure; working hours and shift patterns; sleep; onboard interpersonal relationships; supervisors and shoreside management; general organisational and social support; control and autonomy; uncertainty and insecurity; duration at sea; ship type and size; family; telecommunications; over-commitment; finances; alcohol and smoking; coping strategies; and multiple stressors (the latter theme covers predictive factors examined in studies which were generalised variables consisting of multiple potential stressors). Two studies which discussed mental health interventions for seafarers are discussed separately.

### Sociodemographic and personal characteristics

#### Age

In an analysis of sick leave data, psychological diagnoses affected mostly the youngest age group (30 and under) and decreased with each age category [22]; similarly, the majority of mental illness claims were made by personnel under the age of 40 [23]. Younger staff were also found to experience significantly more self-directed violence (e.g. self-harm, suicidal ideation) [24], depression [25, 26], stress [27], sleepiness [7], distress [28], work-related burnout [29] and mental fatigue [30] than older staff, whilst psychological capital (defined as a set of resources including self-efficacy, optimism, hope and resilience) appeared to increase significantly with age [31]. However, Oldenburg et al. [32] found that age was not significantly associated with emotional exhaustion and Jo and Koh [33] found no association between age and wellbeing. A study of Maritime Academy students found that depression and anxiety were significantly higher in students over the age of 22 [34].

#### Gender

Very few studies considered gender as a predictor of wellbeing because most study populations were predominantly male. Gender was not found to be related to sleepiness [32] or wellbeing [33] although one study of active-duty ship-assigned military personnel found being female was associated with greater odds of screening positive for risk of depression [25].

#### Ethnicity and nationality

Results on ethnicity or nationality as predictive factors of mental health were mixed. More mental illness claims were made by personnel from Europe or the Philippines/Pacific than other parts of Asia [23]. Studies found higher stress in East Asians than South Asians and Caucasians [27] and in South Asians than Caucasian, Mixed, Middle Eastern and Latino/Hispanic personnel [35] and greater physical fatigue in Filipinos than Norwegians [30]. One study [31] found European seafarers had significantly higher psychological capital than Filipinos; another found non-European seafarers had somewhat higher emotional exhaustion than Europeans, but not significantly; nor did they differ in sleepiness [32].

One study found risk of depression was significantly higher in those with Hispanic/Latino ethnicity of either gender, and higher in those of Black ethnicity if they were also female [25]. Tedesco et al. [36] found that mental health symptoms were more prevalent in Italian seafarers than other nationalities. Seafarers from Chinese companies reported higher fatigue than those employed in European countries, regardless of rank or department [37]. However, Oldenburg and Jensen [38] found ethnicity was not associated with subjective stress level.

#### Relationship and parent status

Unmarried personnel were more likely to experience self-directed violence (e.g. self-harm or suicide attempts) [24] and obsessive–compulsive behaviour, paranoid ideation and psychoticism than married personnel [28]. Risk of depression was lower among those in a relationship [25]. Additionally, marital dissatisfaction appeared to be associated with more stress and anxiety [39, 40]. However, one study found wellbeing was not significantly associated with marital status [33]. Seafarers with children reported significantly less emotional exhaustion and less sleepiness than those without [32].

#### Personality characteristics, resilience and psychological capital

Extraverted personality type correlated positively with quality of life, whereas neurotic personality was associated with poorer quality of life [41]. A small number of studies considered the effects of having a more resilient personality, or higher psychological capital, which was associated with lower stress [27, 35], better sleep and lower fatigue [42] and less sleepiness (but only when safety concerns were also low) [43]. One study found that seafarers with high self-efficacy had significantly greater work-related quality of life and less fatigue [44].

#### Prior mental health diagnoses or stressful life events

Prior mental health diagnosis and having at least one recent stressful life experience were both associated with greater odds of screening positive for risk of depression [25]. Seyle et al. [26] also found that more prior experiences of maritime trauma significantly predicted depression; in particular, seafarers who had been held hostage were at greater risk for post-traumatic stress disorder (PTSD), although this was mitigated by perceived utility of pre-departure training.

#### Physical health and injury

High body mass index (BMI) was associated with depression [45] and both underweight and overweight BMI categories were associated with higher global distress [28]. High BMI was a predictor of high stress in surface fleet personnel but not submariners [46]. Conversely, seafarers who reported having a healthy diet on board had better mental health [47]. Personnel who reported poor physical health had higher prevalence of psychoticism, somatisation, depression, anxiety and phobic anxiety [28]. Lefkowitz et al. [45] found that 50% of those with depressive symptoms reported a work injury in the past year compared to 15% of those without; however, it is unclear whether the depressive symptoms or the injury came first.

#### Length of seafaring experience

Longer seafaring experience was significantly associated with lower levels of stress [27] and fatigue [42]. However, longer time in the Navy was associated with higher stress in surface fleet, although not submariners [46] while two studies found no association between experience and emotional exhaustion [32] or wellbeing [33].

#### Rank and role

Whilst many studies found a significant relationship between rank or role and mental health [22–25, 27, 28, 32, 34, 35, 46, 48–54], there was no consistent pattern identifiable to this due to the variety of different roles and outcomes studies. A smaller number of studies found rank/role to be not associated with stress [38] or wellbeing [33].

#### Other socio-demographic and personal characteristics

Risk of depression was lower among those with an undergraduate degree or higher [25]. Wellbeing was not significantly associated with religion [33].

### Physical working and living conditions

The physical work environment was identified as a cause of work-related ill health [22], and in Sliskovic and Penezic’s study [55], the most frequently cited source of job dissatisfaction was the living/working conditions on board (chosen by 35.8% of 530 participants). Participants reported they were negatively affected by noise [33, 56–59]; vibration [33, 57, 58]; ship motion [59]; ambient temperature [56, 58]; poor bedding conditions [56, 59]; restricted living space [33, 54, 59]; ambient light [56, 59]; air pollution [33]; berthing conditions [59]; poor supply, quality and nutrition of food [52, 60]; and poor hygiene [52, 54]. Seafarers with more habitability-related complaints were more likely to report poor sleep [42, 56]; worse mood [56]; greater anxiety and depression [56]; lower job-related affective wellbeing [33]; and greater fatigue [37, 42, 56]. Conversely, other studies found that perception of noise, vibration and movements did not influence emotional exhaustion or sleepiness [7] and disturbance by the physical work environment was associated with only one facet of fatigue (lack of energy) [61].

### Safety

Almost 65% of 917 respondents reported feeling stressed about ship safety [62], whilst 59.5% were concerned about piracy. Bergheim et al. [31] reported a positive association between safety perceptions and psychological capital. Personnel who perceived the organisational safety climate negatively tended to report more mental fatigue, physical fatigue and lack of energy [30] and more sleepiness [43] as well as greater job dissatisfaction and intentions to leave [63].

Also relating to safety, two recent studies focused on the impact of COVID-19 on seafarers. Pesel et al. [64] found that half of their 72 participants did not feel safe doing their job in relation to the pandemic and 60% did not think everything had been done to ensure their health at work in relation to the pandemic. Almost half felt less happy than usual and less able to enjoy their free time, and approximately a quarter reported insomnia, unhappiness and depressive symptoms as a result of the pandemic. In Sliskovic’s study [65], participants reported fatigue and uncertainty relating to the pandemic had a negative effect on their mental and physical health as well as their motivation to work.

### Job demands and pressure

Participants frequently reported high job demands [37] which were perceived as a stressor [28] and associated with fatigue [37, 61] and job dissatisfaction and intentions to leave the industry [63]. Job demands relating to environmental compliance (i.e. work practices relating to the fulfilment of environmental protection requirements on board) were cited as adding to a heavy workload and long working hours, and influencing health and wellbeing in negative ways [66]. Working under time pressure was associated with sleep difficulties, but not acute fatigue [67]. Vigilance demands were associated with sleep problems and had a strong significant effect on chronic fatigue [67], whilst physical work demands predicted high stress in surface fleet personnel but not submariners [46] and psychological demands significantly predicted fatigue [30]. Other specific job demands cited as stressors included too many unnecessary emails from shore office to vessels; difficulties coordinating with different stakeholders such as charters, port operators, and managers; and too many unnecessary and unstructured safety meetings [68]; additionally, in this study being under-staffed was reported to lead to greater job demands.

Inability to disengage with work at the end of a shift significantly predicted stress in submariners [46] and over-commitment was significantly related to work-related burnout [29].

### Working hours and shift patterns

Participants reported long monotonous working hours and inadequate rest [66, 68, 69]; long working hours per day (i.e. more than ten) were significantly associated with emotional exhaustion and sleepiness [32] and those on night shift reported significantly more mental fatigue and lack of energy than those on day shift [30, 49].

Maritime pilots on a four-month rotation system rated their subjective strain as ten times higher than those on one-week rotation systems, and reported more tiredness and more daytime sleepiness between pre- and post-rotation [70]. Incomplete recovery in between work shifts was positively associated with chronic fatigue [67]. Participants typically evaluated 2-watch systems more stressful than 3-watch systems [32]. Job satisfaction and life satisfaction were higher when personnel worked regular, as opposed to irregular, shifts and when there was a favourable ratio between working and free days [71], although there was no relationship between this ratio and mental health. Change of workload and working hours were associated with fatigue [37].

In one study, wellbeing was not significantly associated with overtime work [33].

### Sleep

Seafarers reported that sleep disturbance (caused by constant sleep breaks, working at night and getting up early) was one of the major difficulties they faced onboard [69]. Poor sleep was found to be associated with poorer mental health [47], depression [34], emotional exhaustion and depersonalisation [32] and fatigue [49]. Seafarers with higher than average sleepiness were more likely to experience work-related burnout [29]. In another study, poor sleep quality and sleep disturbance were associated with fatigue in employees of Chinese shipping companies but not European [37].

In Schmied et al.’s study [59], stress was the most common barrier to obtaining sufficient sleep, followed by rotating schedules. In this study, many prioritised other activities over sleep when off duty, often sacrificing sleep for exercising or studying for qualifications. Over half (of n = 22) said stress affected their sleep, often due to work-related concerns and pressures, and many described a state of hypervigilance while on the ship which meant they woke easily.

### Onboard interpersonal relationships

Social cohesion was seen as very important [22]—participants in this study identified several ways of creating social coherence such as allowing dedicated time for interactions at work and during time off; debriefing after demanding shifts; doing activities together outside work hours; common meeting areas and time and space for common activities; and recreational areas such as gyms or lounge areas. Social support from teammates contributed to psychological capital [72], and support in the workplace was the only job resource negatively associated with acute fatigue in one study [67]. The latter study suggested that when time pressure was low, support did not make a difference, but at medium and high levels of time pressure, employees with more support experienced significantly lower levels of chronic fatigue as well as lower inter-shift need for recovery. Additionally, support did not make a difference at high levels of vigilance demands but at low and medium levels of vigilance demands, employees with low social support reported more sleep problems.

Interpersonal relationships were only reported as a source of job dissatisfaction by a minority of participants [55] and levels of bullying and harassment in the workplace were low overall, but more commonly reported by personnel with lower levels of education [73]. Participants in a qualitative study [69] reported that constantly changing crew relationships were stressful; in particular, interacting with people from different cultures and language barriers were negative aspects of their teams. Having people of different ages, education levels and nationalities on board sometimes led to differing views on how work should be managed and how decisions should be made and communicated [22].

### Supervisors and shoreside management

Relationships with immediate managers were considered important for mental health, as were having managers with professional knowledge and experience who understood both the working and living environment on board [22]. In this study, confidence, responsiveness and mutual respect were seen to be the cornerstones of a good relationship between managers and employees. More support from supervisors was associated with less lack of energy, physical exhaustion and lack of moderation [49]; high perception of supervisor support buffered the effects of work-family conflict on physical exertion and physical discomfort but not the mental subdimensions of fatigue.

Supervisors displaying transformational leadership (e.g. providing encouragement, social and emotional support, and motivation) contributed to high psychological capital [72] whilst authentic leadership (defined as leader behaviour a positive work environment and greater self-awareness and self-development) was associated with greater job satisfaction [63].

Perceived lack of care taken by shipboard superiors or the shipping company was associated with emotional exhaustion [32]. When management were perceived to prioritise productivity over safety, seafarers had greater intentions to leave and poorer job satisfaction [63]. Some studies reported negative perceptions of supervisors, suggesting they showed poor communication [66] and inadequate assistance with health problems [54]; many participants felt they had been criticised unduly and were not valued equal to their expectations [73].

Participants in a qualitative study [60] highlighted the importance of organisational justice—that is, an equal and fair work environment—and raised concerns about discrimination and disrespect by some officers; adjustment downwards by officers of exceeded overtime hours; concealment of complains and mistakes by officers; enforcement of different rules by different seniors; infringement of rules by captains; and absence of upward appraisal of seniors by ratings. Participants reported the company did not always understand and appreciate or recognise the efforts of seafarers [68]. Specifically during the COVID-19 pandemic, seafarers reported feeling abandoned by formal organisations in charge of caring for seafarers, describing feeling like prisoners during their extended stay on board despite being classed as key workers [65].

Chinese seafarers reported they were more likely to talk to senior officers or find their own ways to adapt rather than asking shoreside management for help, reflecting a feeling of not being valued by their companies; in contrast, Europeans reported that managers engaged in discussions with them and provided support, a ‘no blame’ culture, performance evaluation meetings, and had developed a system for mitigating fatigue by reducing requirements and workloads [37]. In an interview study [74] crew members felt pressure whenever there was a ship visit by shore management as it could affect their job and promotion prospects. They felt that visits were more about inspection than support, and also reported that visits led to disruptions of their normal working rhythm. Interviews with managers from company officers reinforced this, revealing a sense of distrust on behalf of managers and a focus on surveillance, enforcing safety compliance and disciplinary action rather than provision of support.

### General organisational and social support

A number of papers discussed social support without specifying whether it was from colleagues, management, or non-work sources such as family and friends; these results are discussed within this subtheme. Seafarers with a high level of social support had better health-related quality of life, lower depression, and lower psychosocial stress than those with low or medium support [62]. Kelley et al. [75] found that as social support increased, the indirect effect of stress to problematic alcohol use via depressive symptoms decreased.

Greater instrumental work support was significantly associated with lower stress levels [27, 35] and greater job satisfaction [35]. Bobdey et al. [76] found that personnel living on board a capital ship of the Indian Navy had significantly lower overall perceived social support, appraisal support, self-esteem support and belonging support than those in family accommodation. In Kim and Jang’s study [44], organisational support had an indirect effect on work-related quality of life through a positive effect on self-efficacy and a negative effect on fatigue.

### Control and autonomy

There was feeling among ship crews that they had little control over their job tasks and little participation in decision-making [66], and lack of autonomy was cited as a job stressor [28]. However, more control and greater job autonomy were associated with less lack of energy, less lack of motivation and less sleepiness [61] and less mental fatigue [30]. Job autonomy appeared to act as protective factor for chronic fatigue; at medium and high levels of time pressure, employees with more job autonomy experienced significantly lower chronic fatigue and inter-shift need for recovery [67]. At low and medium levels of vigilance demands, employees with low autonomy reported more sleep problems than those with high job autonomy.

### Uncertainty and insecurity

Participants in several studies reported feelings of uncertainty and insecurity—for example, seafarers on temporary contracts reported multiple forms of work scheduling uncertainty including being deployed at short notice, commencing work on vessels irrespective of whether they had had adequate rest and restoration period at home, often having to board unfamiliar vessels without having familiarisation training, unstable work teams, and a mismatch between actual and expected tour of duty durations due to mandatory tour extensions [77]. In the same study, participants perceived that scheduling and location uncertainties were closely linked with increased risks to safety and wellbeing, which was perceived to be particularly poor when both work location and task uncertainty were experienced together. In Sliskovic’s study of the impact of COVID-19 on seafarers [65], participants reported that not knowing when their onboard work period would end negatively affected their wellbeing. Similarly, mental health was significantly better when there was compliance with the contract regarding changes to ship and home periods [71].

Temporary or insecure contracts were reported by participants to be a factor contributing to depression [78] and job insecurity was the strongest predictor of fatigue [37]. Participants in temporary or apprentice roles reported feeling uncertain about their futures [22] and reported more physical fatigue, mental fatigue and lack of energy than permanent employees [30]. However, in one study [28] temporary workers had higher job satisfaction than regular workers, despite the same study suggesting that instability was a major stressor; the authors suggest this was due to temporary seafarers having more autonomy over their schedules whereas regular seafarers had only short breaks between disembarkation and their next trip at sea.

### Duration at sea

Many participants felt their average length of stay onboard was excessive and that working and living conditions could be improved by having shorter stays on board [38, 79]. One longitudinal study showed that mental health significantly decreased after a long voyage, while somatisation, anxiety and paranoia increased [80], while Hystad and Eid [42] found that longer periods of sea predicted fatigue (but not sleep quality). Shorter duration on board (e.g. two months) was significantly associated with higher job satisfaction and life satisfaction and better mental health [71]. However, other studies found no relationship between duration of time at sea and perceived stress [27] or emotional exhaustion [32].

### Ship type and size

Oldenburg et al. [32] found that seafarers on tankers and passenger liners tended to have higher burnout risk than crews on container and cargo ships. In the same study, those on smaller vessels (2000–5000 gross tonnage) had a higher risk of emotional exhaustion than those on larger vessels, although shipping route had no effect. In Lefkowitz et al.’s analysis of mental illness claims [23], the vessels with the highest reported rates were heavy lift vessels, followed by offshore safety vessels, gas carriers and vehicle carriers. In Tedesco et al.’s study [36], seafarers on merchant ships had greater prevalence of mental health symptoms than those on passenger ships. Seafarers on passenger and cargo ships reported significantly higher levels of fatigue than those in the offshore re-supply industry, and those on ferries had significantly higher fatigue than supply vessels [42].

### Family

Homesickness and loneliness were frequently reported by seafarers [69] along with worries about family [62]. Participants cited loneliness and long separations from home and family as factors causing depressive feelings [78], stress [68] and job dissatisfaction [55]. During COVID-19, reports of loneliness and missing families were aggravated by fear for loved ones because of the pandemic [65]. Work-family conflict was associated with the lack of energy, physical discomfort, lack of motivation and sleepiness dimensions of fatigue [49].

### Telecommunications

Seafarers assigned to worldwide destinations and crew members without children experienced insufficient telecommunication possibilities as work-related strain [38]; in addition, participants reported the cost of telecommunications was too high and internet access on board was limited either by maximum transmittable data volume or internet access duration. When asked how working and living conditions could be improved, the most common answer (among 59.1% of participants) was free telecommunication [79]. However, McVeigh and MacLachlan’s participants reported that internet access was good and was a positive aspect of their life onboard [60]. Additionally, one study found that access to the internet did not have an effect on job satisfaction, life satisfaction or mental health [71], and participants in another study [68] felt that increased access to the internet had reduced socialisation within teams.

### Finances

Over half (55.4%) of Xiao et al.’s [62] participants reported feeling stressed about their financial situation, while only 16.9% felt satisfied about the availability of money for their needs. Conversely, in Sliskovic and Penezic’s study [55], over half of participants cited financial stability and security as their main source of job satisfaction.

### Alcohol and smoking

Work stressors were found to decrease sleep quality which in turn was associated with an increase in alcohol problems [81]; problematic alcohol use was associated with increased stress [75] and depression [25, 75]. One study [36] noted higher voluptuary habits (drinking, smoking, and drug use) among those perceiving high job demands and those boarded on ships docking in a higher number of ports per week. However, Sliskovic and Penezic found that alcohol was consumed less onboard than at home and was not associated with stress or mental health [47], whereas McVeigh et al.’s [68] participants perceived that alcohol prohibition on board reduced socialisation within teams.

### Coping strategies

Participants reported numerous coping strategies to fight fatigue, including trying to be as organised as possible, getting up and walking around regularly keeping busy and active both mentally and physically, and making extra efforts to fight fatigue [82]; in this study, participants tended to prefer behavioural coping strategies when vitality was high and cognitive strategies when vitality was decreased. Coping strategies were also used to adapt to onboard life, such as leisure activities, social wellness (e.g. talking to the crew about their day and forming relationships with others), physical wellness (e.g. food and a healthy regime) and intellectual wellness (e.g. books and learning) [69], as well as using onboard sport and game facilities, communication with family, relaxing and listening to music [79]. However, participants in McVeigh and MacLachlan’s [60] and McVeigh et al.’s [68] studies reported there were fewer opportunities to alleviate stress in recent years due to reduced socialisation and shore leave, and many of Xiao et al.’s [62] participants were dissatisfied with opportunities for leisure activities.

### Multiple stressors

A study of factors affecting psychological status [52] found that the variable ‘working conditions’—a combined measure of crewing strength/number, arrangement of working hours, and work demands—was ranked in top place. Meanwhile Kingdom and Smith [83] used ‘negative occupational factors’ as a variable, combining exposure to physical agents and noise; job demands, control and support; effort-reward imbalance; organisational culture; management of change; leader-member and team-member exchange; bullying; role conflict and ambiguity; training; and perception of stress. Those with higher negative occupational factors had higher stress, anxiety and depression and lower job satisfaction.

### Interventions

Most of the intervention-related papers found in the search were excluded as they focused on seafaring military personnel, with interventions aimed specifically at enhancing combat readiness or improving ability to cope with operational factors. Two papers discussed interventions for non-military seafarers.

McVeigh et al. [68] described the Pilot Resilience Programme, a resilience-training programme aiming to support both on and offshore employees of seafaring organisations, incorporating elements of positive psychology, cognitive-behavioural therapy, neuro-linguistic programming, and leadership training. This intervention involved peer-to peer-training consisting of twelve modules each of 40–60 min duration, covering the topics of resilience, optimism, positive outlook, self-discovery, moving towards goals and being grateful. Although only four of their participants had taken part in the intervention, findings were mixed: some reported positive effects such as strengthened understanding and tolerance of colleagues (although these effects appeared to be short-lived) whilst others felt the personal nature of the programme was an uncomfortable experience. Some indicated a lack of time for implementation of the programme because of how hectic life on board is, suggesting it was seen as just another safety initiative. Participants believed they needed trained facilitators on board to facilitate the programme, and suggested it be adapted to the onboard context (i.e. for people of different ranks, nationalities, and native languages).

Rapoliene et al. [84] compared balneotherapy (the use of natural mineral spring water for prevention and cure of disease, believed to have an anti-stress effect) with music therapy and a control group. The balneotherapy intervention involved a head-out immersion bath with naturally warm highly mineralized geothermal mineral water for fifteen mins daily, five times per week for two weeks. The music therapy intervention involved asking participants to listen to music for twenty minutes with closed eyes and earphones during the same timeframe. The balneotherapy group showed significant improvements compared to both other groups in stress, fatigue, mood and pain.

## Discussion

Although the reviewed studies reported some mixed findings, various potential risk factors for poor mental health of seafarers were identified. The most consistently identified factors for more mental health were younger age; being single; poor physical health; greater exposure to noise and vibration; feeling physically unsafe; high job demands; long hours; night shifts; irregular shifts; poor sleep; poor cohesion within teams; poor perception of supervisor support or shoreside management; lack of social support; lack of job-related autonomy; scheduling uncertainties; long duration at sea; and over-commitment to work. However, as most studies were cross-sectional, some caution is needed in translating the findings into firm recommendations. Nonetheless, the findings of the review identify a number of potential measures which could be incorporated into the workplace to improve seafarers’ mental health.

In terms of personal and socio-demographic characteristics, poor mental health and wellbeing appear to be associated with younger age, being single, and being in poor physical health. It is unclear why younger personnel might experience poorer mental health, but one possibility is that, as their jobs are relatively new compared to older employees, they experience less job autonomy and job security—both factors which affect mental health according to this review; their inexperience may also make it more difficult to cope with high job demands and may make decision-making more stressful. This supports previous research highlighting the difficulties faced by young employees during their transition into employment and the challenges of adapting to demanding work whilst overcoming inexperience and developing life skills [85, 86]. It is also possible that the comparative resilience of older employees could be due to the healthy worker effect in that more resilient staff remain in a job for years whereas less resilient staff leave earlier [87].

It is also unsurprising that being single was a risk factor for poor wellbeing, as being in a (supportive) relationship has frequently been associated with better mental health [88]; additionally, social support appears to reduce occupational stress [89], so the support received from a partner and the ability to share concerns about work with them is likely to be beneficial. The relationship between physical and mental health is also well-established [90] and so it is perhaps to be expected that seafarers in poor physical health also reported more mental health problems. We suggest it would be beneficial if managers paid particularly close attention to seafarers in these potentially vulnerable groups (young, single, poor physical health)—and particularly any seafarers who meet all three of those criteria—as they may need extra support and occupational health advice might be warranted.

Managers may also benefit from training in how to recognise symptoms of mental health problems in their staff. Managers leading by example and being open and responsive to discussions with their crew would be beneficial to younger, less experienced personnel. Additionally, a peer support ‘buddy system’ or mentoring system may be beneficial for less experienced staff. Encouraging good relationships between employees may provide an extra level of social support which could be beneficial for single employees.

Providing education on how to maintain a healthy lifestyle on board, and making staff aware of the psychosocial factors contributing to poor health, could also be beneficial [4]. As unhealthy BMI appeared to be related to poor mental wellbeing in this review, healthy eating and exercise habits should be encouraged where possible, and the food available on board should be high in quality and nutrition. Having a fitness room on board could encourage exercise to improve health and also encourage socialisation with team members; exercise could also benefit maritime personnel’s mental wellbeing [91, 92].

Exposure to poor physical conditions (such as noise, particularly high or low temperatures, and vibration) is unavoidable in seafaring professions. However, steps could be taken to improve the physical environment such as reducing noise in cabins, which could improve seafarers’ psychological capital [72]. Oldenburg et al. [1] also recommend reducing exposure to noise in accommodation, recreational and catering facilities, in addition to increased education about the effects of noise exposure and instructions on the use of noise protection equipment. We agree that these could be effective ways of reducing exposure to noise. Additionally, reducing durations of stay on board and reducing long working hours would limit the duration of exposure to negative physical conditions.

Feeling unsafe on board also appears to be associated with poor mental health, which is unsurprising as research on employees in potentially dangerous roles has frequently suggested that perceptions of danger and threat to safety significantly predict poor mental health [93]. We suggest that it may be useful for maritime personnel if their managers emphasise that safety is taken seriously and reassure their staff about the safety procedures in place. Managers could also promote appropriate safety behaviour, leading by example to provide a role model for employees. There is evidence that leadership is an important factor in promoting safety behaviour in the workplace, with a more positive safety culture associated with managers having a clear vision for safety, acting as role models, showing concern for employee welfare, communicating clear safety standards and goals, and acknowledging positive safety behaviours [94]. Taking steps to improve general psychological wellbeing onboard could also lead to better safety behaviour, as there is some evidence that being resilient and having strong psychological capital promotes better safety behaviour [72].

Good relationships between team members can promote better mental health [93, 95, 96] and positive relationships onboard should be fostered. Leaders within maritime organisations should foster an inclusive culture, encouraging personnel to embrace the different nationalities, cultures and personalities on board rather than discriminate because of them. Indeed, the National Institute for Health and Care Excellence (NICE) guidelines for promoting employee wellbeing [97] highlight the importance of eliminating discrimination in the workplace. Therefore, maritime organisations should have clear policies around discrimination; any reports of bullying, discrimination or harassment should be thoroughly and swiftly investigated; and steps taken to eliminate a negative workplace culture reassure staff of their commitment to a safe, inclusive workplace for all employees. Having social events on board could improve relationships between team members, and also reduce the boredom and loneliness often experienced by seafarers.

Good management and leadership appear to be important. This supports previous research suggesting that leadership strongly predicts mental health and wellbeing of staff in various occupational groups, including the military [95], healthcare workers [98] and disaster relief workers [93]. Good leadership not only affects mental health but also helps to shape the work safety climate and can lower the risk of workplace accidents [99]. Oldenburg et al. [7] recommend improving superiors' communication and leadership skills in order to improve seafarers’ mental health; we recommend that both on-board managers and shoreside managers should receive training in good leadership skills.

Long hours, night shifts, irregular shifts and lack of sleep all appear to negatively affect seafarers’ mental health, in addition to high job demands. Increasing crew numbers would be the most obvious solution to each of these issues. Carter [4] emphasises the importance of ensuring that crewing levels are sufficient to handle the required tasks, and we suggest that increasing crewing levels would share the workload (and hours) out among more staff allowing seafarers longer rest periods.

A systematic review of occupations with limited wake shift work schedules including maritime personnel and long-haul train drivers [100] suggests that shorter time at work, more frequent rest breaks, shifts that start and end at the same clock time every 24 h, and work shifts commencing in the daytime are optimal. Having more staff would allow for shorter shifts and more frequent rest, and having regular shifts may also help reduce the negative feelings associated with uncertainty. Working excessively long hours puts staff at risk of fatigue and can endanger the crew: sleep deprivation has been shown to have a negative effect on judgment and decision-making [101], which could have catastrophic consequences in a hazardous environment such as a vessel at sea. Limiting work hours and ensuring staffing levels are sufficient is therefore extremely important.

It appears that long duration at sea can reduce seafarers’ wellbeing. This is unsurprising as the longer personnel are on board, the longer they are exposed to any other stressors which may be present in their workplace such as poor physical conditions, lack of sleep, high demands or poor relationships with team members or supervisors. Carter [4] recommends reviewing the periods of maximum continuous service at sea, and Oldenburg et al. [1] recommend shortening the duration of shipboard stay. Shorter durations of stay on board would limit the exposure to other onboard stressors and also reduce the time separated from families and offshore support networks.

Lack of job-related autonomy, and uncertainties around scheduling, appeared to leave many seafarers feeling they lacked control over their work. This supports the large body of literature suggesting that lack of autonomy and control in the workplace is associated with poorer wellbeing in various sectors [95, 102, 103]. Job-related autonomy could be improved by allowing employees greater control over their own tasks and greater involvement in decision-making; however, we note that interventions aimed at improving autonomy have had mixed results in terms of succeeding in enhancing autonomy [104] and so further research on how to achieve this would be beneficial. There was also some evidence that job insecurity may affect wellbeing; this has also been noted in other professions, with job insecurity related to poorer wellbeing [105] and lower happiness [106], and improving the long-term security of seafarers’ employment would be useful in improving their wellbeing [4]. Feelings of job insecurity could potentially be improved by participation in further training to improve perceived employment prospects and by employers supporting their employees’ training through financial support and allowing them adequate time to participate in the training [107].

There was some evidence that over-commitment to work—i.e. finding it hard to disengage from work outside of work hours—was negatively associated with mental health. Previous research has associated over-commitment with exhaustion [108]. Increasing opportunities for recreational activities—which may be easier if work hours are reduced—and promoting social events on board could potentially help staff to disengage. This would also be helpful in strengthening social support networks within the workplace.

We acknowledge that seafaring organisations may not always have the means to implement all of the recommendations provided, and that even if suggestions are implemented personnel will still have some level of occupational stress purely due to the nature of their work. For this reason, we suggest supervisory personnel should receive training in how to identify and manage pressure/stress in others (and themselves). This suggestion is supported by Oldenburg et al. [1] who recommend training for ship officers on how to prevent and manage stress.

Literature on the effectiveness of interventions to improve seafarers’ psychological wellbeing was scarce. Only two relevant intervention studies were included in this review, and as both assessed very different interventions, it is not possible to draw any conclusions or recommendations from our review of two studies. Our perception from the papers we saw during the screening process involved in this review is that the majority of interventions designed for those working at sea are aimed specifically at military populations, who face the additional stressor of potential combat. Whilst some of these interventions are not immediately applicable to civilian maritime organisations [109] they could perhaps be adapted or some elements carried over to civilian interventions. We would therefore recommend that future reviews of any intended stress-management interventions could be done by extracting all data relevant to improving the wellbeing of a seafaring population, and assessing which aspects of the interventions would be appropriate for civilian seafaring organisations, although we anticipate that this approach may be somewhat challenging.

In terms of future research, randomised controlled trials would be needed before determining how feasible and effective it would be to provide interventions to alter the identified risk factors and regression analysis to assess the strength of association between variables would also be useful. The majority of the studies reviewed were quantitative and so more qualitative research would be helpful in order to improve understanding of the lived experiences of seafarers. The qualitative research we identified tended to rely on interviews; ethnographic studies or focus groups could also be used. Another potential avenue for research might be Online Photovoice [110], allowing seafarers to tell their stories through photographs in order to increase knowledge of what their experiences are like and promote dialogue around the issues they face.

### Limitations

There are a number of limitations, both of the papers included in this review and the review process itself, which must be considered. First, in terms of the papers included, quality was mixed and indeed sometimes poor. Most relied on convenience sampling and many failed to consider non-responders, meaning that the picture provided of seafarers’ stressors may not necessarily be representative of the seafaring population as whole. Some caution must therefore be taken in generalising these results.

In terms of limitations of this review, the decision to limit the search to English-language papers means that potentially relevant studies published in other languages were excluded. Future reviews may consider not limiting by language, and translating foreign-language papers, in order to provide a full global picture of the factors that affect seafarers’ wellbeing. Searches were also restricted to peer-reviewed journals; this review may therefore be subject to publication bias and future reviews could consider reviewing grey literature to compare the findings of unpublished research to the findings of this review. We must also acknowledge the possibility that papers meeting our inclusion criteria may have been missed, due to the search strategy used or the databases searched; reviews using broader search terms or a wider variety of databases may have uncovered additional papers. Hand-searching the reference lists of included papers would also have been beneficial in identifying additional papers.

Finally, it needs to be noted that the searching, screening, data extraction and data synthesis processes were all carried out by one author. Although any concerns or queries were discussed with the other study author, it would strengthen the validity of this review if a sample of studies underwent double screening and data extraction.

### Recommendations

Overall, the results of this review identify the following potential recommendations for improving the mental wellbeing of seafaring personnel:

- Training for senior staff/supervisors in how to recognise mental health problems in their staff
- Increased monitoring of the wellbeing of all staff, particularly those in vulnerable categories such as younger, single staff with poor physical health
- Senior staff should act as role models for younger, less experienced staff, who could also potentially benefit from a mentor or buddy system
- Increase awareness and education about how to maintain a healthy lifestyle on board
- Improve nutrition available on board
- Increase quality and availability of exercise facilities/equipment on board
- Reduce noise in cabins
- Increase awareness and education about the effects of noise exposure and importance of using noise protection equipment
- Increase crewing levels to spread the workload out among a greater number of personnel; this should also reduce durations of stay on board and reduce long working hours
- Managers should ‘lead by example’ in terms of promoting safety behaviour
- Reassurance from managers that safety, both psychological and physical, is taken seriously
- Managers should encourage strong relationships between team members, perhaps via social events on board
- Managers should foster an inclusive culture and reassure employees that any reports of negative workplace behaviour will be taken seriously and dealt with quickly and appropriately without inappropriate impacting on individual’s careers
- Training in leadership and management skills for both on-board and shoreside managers including being aware of potential indicators or poor mental health and fostering a broad understanding of how to support staff mental health
- Make use of shorter, more regular shifts where possible
- Allowing employees to be involved in organisation decision-making processes where appropriate
- Allowing employees more control over their tasks
- Encouraging employees to participate in further training when desired
- Increased opportunities for recreational activities
- Provision of training in coping skills and stress management.

## Data Availability

The study was a systematic review and the data that formed the findings was extracted from the articles listed in Table 1.

## Abbreviations

AXIS: Appraisal tool for cross-sectional studies
BMI: Body mass index
CASP: Critical Appraisal Skills Programme
COVID-19: Coronavirus disease-19
ILO: International Labour Organization
ISWAN: International Seafarers’ Welfare and Assistance Network
NICE: National Institute for Health and Care Excellence
PTSD: Post-traumatic stress disorder
